# Development of selected bacterial groups of the rectal microbiota of healthy calves during the first week postpartum

**DOI:** 10.1111/jam.14484

**Published:** 2019-11-05

**Authors:** K. Schwaiger, J. Storch, C. Bauer, J. Bauer

**Affiliations:** ^1^ Department of Animal Sciences Chair of Animal Hygiene School of Life Sciences Weihenstephan Technical University of Munich Freising Germany; ^2^ Department of Quality Assurance and Analytics Bavarian State Research Center for Agriculture Freising Germany; ^3^ Present address: Landratsamt Donau‐Ries Veterinäramt Donauwörth Germany

**Keywords:** Calf, gut microbiota, *Enterobacteriaceae*, enterococci, lactobacilli, *Lactobacillus reuteri*

## Abstract

**Aims:**

The intestinal microbiota of newborn calves is largely unexplored even if it is of great significance for their future health. Therefore, the aim of the study was to gain a better insight into the development dynamics of certain bacterial groups during the first week of life.

**Methods and Results:**

Faecal samples of healthy Simmental calves (dual‐purpose breed; *n* = 80), bottle fed and raised in a dairy farm were taken immediately after birth and at 6/12/24/48/72/168 h (h) after birth. Samples were analysed using cultural, biochemical and molecular–biological methods. The aerobe, anaerobe, *Enterobacteriaceae* and *Enterococcus* counts of healthy calves increased significantly between 6 and 24 h postpartum (*P* <0·05). Apart from the anaerobes, bacterial counts decreased after reaching a plateau at 24–48 h. *Enterococcus faecalis* was detected in significantly higher counts compared to *E. faecium* (*P* <0·05). Lactobacilli developed more slowly and increased until day 7 after birth to a mean value of 6·8 × 10^7^ CFU per g. MALDI‐TOF analysis of 2338 lactobacilli isolates resulted in 36 different species.

**Conclusions:**

*Lactobacillus reuteri* became the most common *Lactobacillus* sp. during the first week of life.

**Significance and Impact of the Study:**

This fact seems to be very important for the calf’s intestinal health because *L. reuteri* is known to show in vitro bactericidal effects against bacterial pathogens and anti‐infective activities against rotaviruses and *Cryptosporidium parvum*.

## Introduction

One of the most important events during and after the birth of mammals is the colonization of the skin and mucous membranes with micro‐organisms: a germ‐free organism is suddenly confronted with a high variety of microbes,for example, bacteria, viruses and fungi. Most of the previous studies were focused on pathogens (e.g. *Salmonella enterica*, *Escherichia coli*, rotavirus, coronavirus *Cryposporidium parvum*) that can endanger the newborn (Cho and Yoon, 2014; Gomez and Weese 2017). However, there is increasing evidence that the colonization of the mammalian gastrointestinal tract plays a crucial role in the development of the mucosal immune system and influences the growth and performance of the individual (Schwarzer *et al.* 2016; Clavel *et al.* 2017). In summary, the evolution of the microbiota has great significance for the health of the newborn.

The colonization of the gastrointestinal tract is a complex process influenced both by the host and by the microbes. First systematic investigations on the development of bacteria in the gut of newborn calves were carried out in the early 1960s (Smith and Crabb 1961; Smith 1965). Although there are some publications dealing with the calf’s intestinal microbiome, there are only a few studies that cover the development within the first week of life (Takino *et al.* 2017; Song *et al.* 2018). This is surprising, considering that enteric infections in neonatal calves are amongst the major causes of calf mortality during the early stage of life (Bendali *et al.* 1999). Moreover, precisely in this period the course is set for the overall development of the calf. Even if the animals survive, negative effects on the subsequent performance (body weight gain, pelvic height growth) of cattle are detectable (Donovan *et al.* 1998).

Due to these circumstances, we studied the development of the rectal microbiota of calves during the first week of life. For this purpose, we linked quantitative cultural methods with MALDI‐TOF analyzes. This approach allowed the representation of the postnatal development of certain bacterial genera – especially *Lactobacillus* – at species level.

## Materials and methods

### Animals

The study included 150 newborn Simmental calves (66 female, 84 male) reared in 13 Bavarian farms (cows/farm: 55–165). Since all the farms were supervised by the same feeding consultant, calf’s‐management and feeding designs were very similar during the first week of life. In all farms, the calves were placed within 24 h postpartum in so‐called calf‐igloos or weather‐protected individual boxes in the outdoor area. Colostrum was administered as soon as possible – on average 1·7 h postpartum. On average, the calves consumed 1·9 liters (0·5–4·0 l) of colostrum at the first feeding. In addition, colostrum was fed for the first 5 days (3–4 times/day), after which the calves received a milk replacer. The cows were not treated with antibiotics during the perinatal period.

The health of the calves was monitored throughout the study period by conventional clinical investigation (Stöber 2012). Faecal samples were collected immediately after birth (meconium) and 6, 12, 24, 48, 72 (3 days) and 168 (7 days) h postpartum for microbiological investigations. Animals showing signs of disease (e.g. diarrhoea, pneumonia) were excluded from data analysis. Overall, complete data sets of 80 healthy calves were evaluated. Due to technical reasons differentiation up to species level was only possible for the isolated strains of 69 healthy calves.

### Sampling

The anal region of the calves was cleaned and disinfected. Then, an industrial‐clean glove was put on and the faeces were removed with a finger directly from the calf's anus. The glove was pulled inside out, knotted and placed in a freezer bag. This procedure should ensure that contamination is largely avoided. The samples were stored at 4–8°C for a maximum of 48 h until further investigation.

### Microbiological examinations

Total aerobe and anaerobe colony forming units (CFU) in the faecal samples were quantified by the reference spatula method on Standard I Nutrient agar (Merck, Darmstadt, Germany) containing 7% defibrinated sheep blood and Schaedler agar with 5% sheep blood (Fiebig, Idstein‐Niederauroff, Germany) respectively (Gedek, 1974). In brief: Faeces (1 g) were diluted in 9 ml physiological saline and a dilution series (factor 1 : 10) was prepared. Of the dilutions, 0·1 ml were pipetted onto the different growth media (see below); this procedure ensures a detection limit of 10^2^ living bacteria/g faeces. The criteria for counting the different bacterial groups were growth on selective agar plates as well as morphology and colour of the colonies. In addition, representative colonies were analysed by microscopical, biochemical and/or MALDI‐TOF methods (see below).

The number of *Enterobacteriaceae* was determined using Gassner agar (Merck, Darmstadt, Germany), lactobacilli using LAMVAB‐agar (Hartemink and Rombouts 1999) and enterococci using citrate azide tween carbonate (CATC) agar (VWR, Darmstadt, Germany), the latter without counting colourless colonies. The inoculated agar plates were incubated aerobically (blood agar, Gassner agar, CATC agar) or anaerobically (Schaedler agar, LAMVAB‐agar) for 48 h at 37°C.

*Escherichia coli*, *Enterococcus faecalis* and *Enterococcus faecium* were biochemically characterized and identified as follows: *E. coli* was isolated on Gassner agar and confirmed by the production of β‐D‐glucuronidase and by the criterion of metabolizing sorbitol (Fluorocult agar, Merck, Darmstadt, Germany). Suspicious lactose‐positive colonies not matching these criteria were investigated by API 20E (Biomerieux, Nürtingen, Germany). Lactose‐ and indole‐ negative strains were tested with antiserum *Salmonella* Omnivalent (Sifin, Berlin, Germany) for agglutination. Other *Enterobacteriaceae* species were identified using API 20 *E. faecalis* and *E. faecium* were isolated on CATC‐Agar and confirmed biochemically by testing their metabolism of xylose, mannitol, arabinose and sodium‐pyruvate (Bejuk *et al.* 2000).

For the identification of *Clostridium* spp., bacteria growing under anaerobic conditions on Schaedler‐agar were subcultured both under aerobic and anaerobic conditions to exclude facultative anaerobic bacteria. The remaining obligatory anaerobes were identified at genus level as *Clostridium* spp. by means of micro‐morphological (Gram+/−, rods, spores) and biochemical criteria (catalase−, oxidase−, indole+/−).

Bacteria of the genus *Lactobacillus* on LAMVAB‐agar were identified on genus level by microscopic (Gram+, rod, no formation of spores) and biochemical criteria (catalase−, oxidase−, indole+/−). The determination of the species was carried out by MALDI‐TOF‐MS (Bruker Microflex™ LT; Bruker, Billerica, MA). Briefly, spectra from each isolate were obtained in accordance with the manufacturer’s instructions using fresh and pure cultures. A small amount of culture material was transferred with an autoclaved small wooden stick onto a 96‐spot polished steel target plate and overlaid with one microlitre of α‐cyano‐4‐hydroxy‐cinnamic acid. After drying at room temperature, data acquisition was performed using a Bruker Microflex™ LT equipment (Bruker) and the Biotyper Real Time Classification software (Bruker Daltonics, Bremen, Germany).

### Statistical analysis

For statistical analysis, CFU counts were logarithmized (log_10_). Results below the detection limit of 10^2^ CFU per ml were set to ‘0’. When two groups were compared, the *t*‐test was performed when the data followed a normal distribution and the variance of the variables was the same; otherwise the Mann–Whitney U test was applied. When comparing several groups, analysis of variance (anova) or Kruskal–Wallis test were used; in addition, individual groups were then compared in pairs performing the Holm–Sidak and Dunn test respectively. To evaluate the scattering of the data points, the 25 and 75% quantiles (Q1; Q3) were calculated and divided by the median for standardization.

## Results

For analysis of the development of culturable microbiota during the first 7 days of life, 560 samples of 80 healthy calves were examined. For further analysis, 4395 bacterial isolates were differentiated more precisely.

### Aerobes

**Figure 1:**
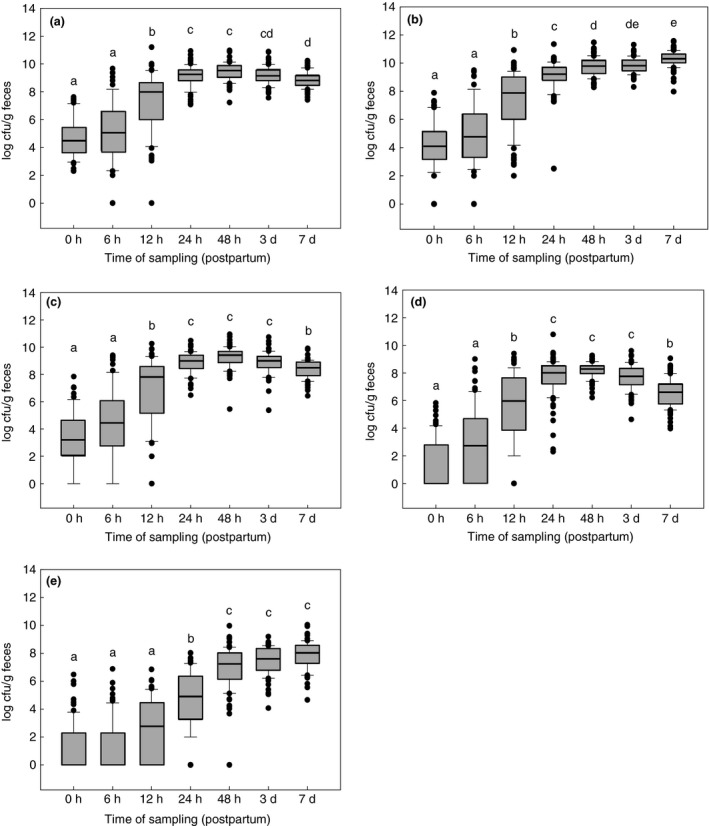
Development of different groups of bacteria in faeces samples of healthy calves (*n* = 80) during the first week of life. (a) aerobes; (b) anaerobes; (c) *Enterobacteriacea*e; (d) enterococci; (e) lactobacilli. 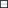 50% of the values, 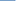 median, 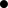 extreme values, 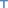, 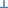 whiskers, a–e different letters indicate significant differences between all times of sampling (anova, Holm–Sidak‐test; *P* < 0·05). [Colour figure can be viewed at http://wileyonlinelibrary.com]

The development of the aerobic microbiota is shown in Fig. 1a. The bacteriological examination of faeces samples revealed total germ counts between 1 × 10^2^ (limit of detection) and 1·6 × 10^11^ CFU per g. Bacteria were detected in all meconium samples (minimum 2 × 10^2^ CFU per g faeces), whereas three 6‐h samples and one 12‐h sample were below the detection limit. The maximum value of a meconium sample was 4·2 × 10^7^ CFU per g. Significant increases (*P* < 0·05) in germ counts were seen between 6 h and 12 h and between 12 and 24 h. After reaching a peak at 48 h, the number of germs declined again significantly between day 3 and 7 (*P* < 0·05).

### Anaerobes including clostridia

Determining the total counts of anaerobes, five meconium samples and three samples taken 6 h postpartum were under the limit of detection (Fig. 1b); one meconium sample contained a maximum number of 7·8 × 10^7^ CFU per g. The maximum value was 3·8 × 10^11^ CFU per g in a sample collected on the 7th day of life. In contrast to the aerobic counts, anaerobic total counts continued to increase until the 7th day of life. Immediately after birth, clostridia could only be detected in one calf. The percentage of clostridia‐positive calves increased to 53·6% (*n* = 37) at 48 h, and then dropped to 18·8% (*n* = 13) until day 7 (data not shown).

### Enterobacteriaceae

**Figure 2:**
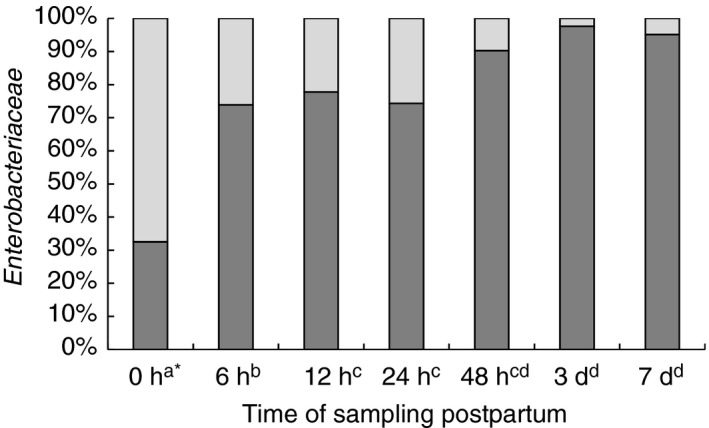
Evolution of the percentage of *Escherichia coli* in the average total number of *Enterobacteriacea*e in the calf intestine during the first week of life. 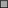 *E. coli*, 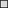 other *Enterobacteriaceae*. ^*^Different superscripts at sampling times indicate a significantly different proportion of *E. coli* (Peto and Peto 1972; *P* < 0·05).

*Enterobacteriaceae* could not be detected in 19% of the meconium samples; in addition, in 18% of the 6‐h samples and in 4% of the 12‐h samples no bacteria of this family were detectable. The highest number of *Enterobacteriaceae* was found to be 8·9 × 10^10^ CFU per g in a sample taken 48 h postpartum. According to Fig. 1c, the maximum viable numbers were detected at 24‐h postpartum which remained constant until a reduction was observed on day 7. When *Enterobacteriaceae* were differentiated judging their ability to utilize lactose, it was noted that lactose‐negative bacteria predominated immediately after birth. However, after just 6 h, they only accounted for <20% of all *Enterobacteriaceae*, dropping to 0·2% after 48 h. Overall, the absolute number of lactose‐negative bacteria was relatively low (on average 10^2^ CFU per g). Growing on plate‐count agar, most of them were yellow pigmented and were identified by API in about 80% of the investigated colonies as species of the genus *Pantoea*; occasionally, *Pseudomonas*, *Hafnia*, *Serratia*, *Proteus* or lactose‐negative *E. coli* were analysed. However, *Salmonella* were not detected by biochemical and serological methods. All lactose‐ and indole‐positive, as well as oxidase‐negative bacteria, which showed fluorescence on Fluorocult agar, were classified as *E. coli*. All bacteria that were lactose‐negative but otherwise met the above criteria were also identified as *E. coli* by the API test system. Serological testing of fluorescence‐negative bacteria for EHEC was negative in all cases. Figure 2 shows the proportion of *E. coli* in the average total number of *Enterobacteriaceae*. While the proportion of *E. coli* directly after birth was only 33%, it increased within 6 h to 74%, and finally accounted for more than 90% after 48 h. Random examinations by API identified, in addition to *E. coli* and the already mentioned lactose‐negative bacteria, other species such as *Klebsiella* spp., *Enterobacter* spp, and *Escherichia fergusonii*.

### Enterococci

**Figure 3:**
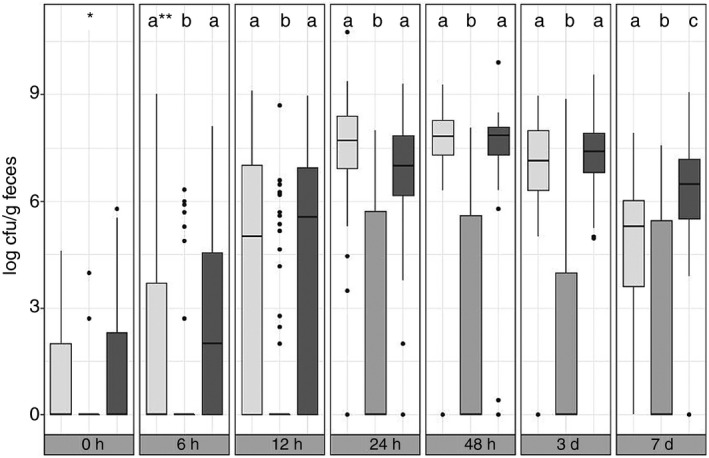
Development of different enterococci groups in faeces samples of healthy calves (*n* = 69) during the first week postpartum. 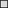 *Enterococcus faecalis*, 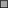 *Enterococcus faecium*, 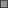 *Enterococcus nonfaecalis/nonfaecium*; *Data not sufficient for statistical analysis; **Columns with different superscripts at the same sampling time differ significantly (Peto and Peto, 1972; *P* < 0·05).

Enterococci, like *Enterobacteriaceae*, could not always be detected from the beginning; 57/36/9% of the samples obtained at time points 0/6/12 h postpartum contained culturable counts below the detection limit. The highest value (6·1 × 10^10^ CFU per g) was measured in a sample obtained 24 h postpartum. The course of development of enterococci was similar to the course of the aerobic counts (Fig. 1d). Based on their sugar metabolism, the enterococci were further grouped into *E. faecium*, *E. faecalis* and *E. nonfaecalis/nonfaecium*. It was noticeable that the development of the *E. faecalis* and the *E*. *nonfaecalis/nonfaecium* group was relatively similar both to each other and to the total number of enterococci (Fig. 3). Only on the 7th day after birth the *E*. *nonfaecalis/nonfaecium* group (average 1·5 × 10^6^ CFU per g faeces) was significantly overrepresented compared to *E. faecalis* (average 2·9 × 10^4^ CFU per g faeces; *P* < 0·05)). On the other hand, *E. faecium* was rarely isolated from the faecal samples and averaged less than 10^2^ CFU per g faeces.

### Lactobacilli

Lactobacilli tended to develop more slowly than the other groups of the investigated germs: 66% (*n* = 53) showed no growth on LAMVAB‐agar, neither in the meconium samples nor in the 6 h samples. Thirty‐six per cent of the 12‐h samples, 9% of the 24‐h samples and one 48‐h sample showed also no growth for lactobacilli. A significant increase in the number of germs was also recorded between 12 h and 24 h and between 24 h and 48 h (*P* < 0·05; Fig. 1e). In addition, the scattering of data points was reduced only from 48‐h postpartum in comparison to earlier time points. Similar to the anaerobes, the lactobacilli showed a continuous, though not significant increase in germ counts until the 7th day of life. The maximum of 1·1 × 10^10^ CFU per g faeces was measured in a 7‐day sample.

A total of 2338 isolates selected from LAMVAB‐agar were analysed using MALDI‐TOF mass spectrometry. Table 1 lists the identified species (*n* = 2075) and the number of corresponding isolates.

**Table 1:** MALDI‐TOF‐MS identification of lactobacilli (*n* = 2075) isolated from LAMVAB‐agar

Species	Isolates (*n*)
*Lactobacillus reuteri*	476
*Lactobacillus paracasei*	401
*Lactobacillus murinus*	240
*Lactobacillus salivarius*	206
*Lactobacillus fermentum*	150
*Lactobacillus mucosae*	116
*Lactobacillus plantarum*	115
*Lactobacillus rhamnosus*	98
*Lactobacillus parabuchneri*	96
*Lactobacillus brevis*	38
*Pediococcus acidilactici*	22
*Lactobacillus saerimneri*	20
*Lactobacillus buchneri*	14
*Lactobacillus curvatus*	10
*Lactobacillus agilis*	8
*Lactobacillus ingluviei*	8
*Pediococcus pentosaceus*	8
*Lactobacillus casei*	6
*Lactobacillus coryniformis*	6
*Lactobacillus oris*	6
*Lactobacillus kefiri*	5
*Lactobacillus diolivorans*	3
*Lactobacillus paraplantarum*	3
*Lactobacillus pentosus*	3
*Lactobacillus farciminis*	2
*Lactobacillus nantensis*	2
*Lactobacillus parakefiri*	2
*Leuconostoc lactis*	2
*Leuconostoc pseudomesenteroides*	2
*Lactobacillus fructivorans*	1
*Lactobacillus kimchii*	1
*Lactobacillus manihotivorans*	1
*Lactobacillus perolens*	1
*Lactobacillus ruminis*	1
*Lactococcus garviae*	1
*Lactococcus lactis*	1

Table 2 shows the 10 most frequently detected *Lactobacillus* species and their corresponding bacterial counts. Frequently, no lactobacilli could be detected during the first sampling times. *L. brevis* and *L. plantarum* were most commonly detected at 0 h and 6 h, respectively, but *L. brevis* could not be detected in the further course of the investigation. *Lactobacillus parabuchneri* and *L. paracasei* increased steadily after 6 h, but then declined (not significantly) at the 7th day after birth. At 48 h, *L. murinus* predominated among the lactobacilli population of healthy calves but was overtaken by *Lactobacillus reuteri* at the 3rd day; the latter increased up to the 7th day to 8·39 log CFU per g faeces. In addition to *L. reuteri*, also *L. fermentum, L. mucosae, L. rhamnosus* and *L. salivarius* showed an increase until day 7, albeit less continuously and at lower levels (up to 7·98 log CFU per g faeces).

**Table 2:** Occurrence of dominant *Lactobacillus* spp. (mean value; log_10_ CFU per g) in faeces samples of healthy calves (*n* = 69)

Species	Time of sampling postpartum						
	0 h	6 h	12 h	24 h	48 h	3 days	7 days
*L. brevis*	4·74^a,^*	2·33^a,b^	2·57^a^	4·53^b^	0·00^c^	0·00^c^	0·00^c,d,f^
*L. fermentum*	0·00^a^	0·00^a^	0·00^a^	4·64^b^	6·40^a,b,e,f^	6·74^b,d^	7·59^b,c^
*L. mucosae*	0·00^a^	3·91^a^	1·45^a^	4·73^b^	6·58^b,c,e,f^	6·58^c,d,e^	7·98^b^
*L. murinus*	0·00^a^	0·00^a^	2·22^a^	6·18^b,c^	8·30^a^	7·68^a,b,g^	5·57^c,e,f^
*L. parabuchneri*	0·00^a^	3·16^a^	3·55^a^	5·49^b^	6·36^b,c,d^	6·50^c,d^	6·24^c,f,g^
*L. paracasei*	0·00^a^	4·47^a^	4·82^b^	6·52^a^	7·09^a,d^	7·24^b,e,f^	6·63^b,c^
*L. plantarum*	4·22^b^	5·38^b^	3·73^a^	5·30^b^	5·96^c,e^	6·81^c,d^	6·08^c,f.g^
*L. reuteri*	1·17^a^	0·76^a^	4·40^a^	4·56^b.d^	7·50^a^	7·69^a^	8·39^a^
*L. rhamnosus*	0·74^a^	0·00^a^	3·92^a^	3·70^b^	6·05^b,c,d^	6·48^c,d,f^	7·57^b,e,g^
*L. salivarius*	0·00^a^	1·98^a^	3·68^a^	6·27^b^	6·87^a,d,f^	6·64^d,f,g^	7·87^b,f^

* Numbers of a column, characterized with a different superscript differ significantly (*P* < 0·05)

## Discussion

In the age of next generation sequencing (NGS), it may seem antiquated to conduct such a comprehensive study using conventional cultural techniques. In fact, there is no doubt that 16S rRNA‐based NGS is a powerful tool to investigate the gastrointestinal microbiota. However, NGS studies mostly characterize the composition of the microbiota at the level of Phyla or Genera, and quantitative differences are usually shown as relative abundances. Since an accurate conversion of abundances to cell numbers is still not solved (Bonk *et al.* 2018), NGS allows only a limited quantitative statement. The objectives of our study were: (i) to record the kinetics of the culturable microbiota during the first week of life, and (ii) to demonstrate the dynamics of important bacterial groups. For example, by combining quantitative cultural techniques with MALDI‐TOF, we gained new detailed insights into the development of dominant *Lactobacillus* species in new‐borne calves (Table 2).

The data of 80/69 calves from 13 farms were summarized and analysed in total. Although it is known that a ‘farm‐effect’ on the intestinal microbiota of calves exists (Weese and Jelinski, 2017), such an evaluation was waived; the reasons for this approach were the relatively low number of calves per farm and the associated low explanatory power of the results. In addition, an evaluation according calves’s management, feeding design or feed, was also omitted, since these criteria were very similar in all farms.

The aerobic and anaerobic bacterial counts initially increased largely parallel and reached maximum values of 10^11^ CFU per g faeces. Malmuthuge *et al. *(2012) also reported a total bacterial count of 10^11^ CFU per g via qPCR in the colon content of calves at the age of 3 weeks. Likewise, Dowd *et al. *(2008) detected bacterial counts of up to 10^11^ CFU per g in faecal samples of adult cows. The number of strict anaerobes (e.g. *Clostridium* spp.) normally exceeds that of facultative anaerobes (e.g. *Enterobacteriaceae*) by a factor of 100 (Guarner 2006; Dowd *et al.* 2008). This observation coincides with the results of the present study: While the anaerobes continued to increase to an average of 10^10^ CFU per g faeces until the 7th day after birth, the aerobic bacteria, the enterobacteria and the enterococci decreased statistically significant again to below 10^9^ CFU per g after reaching a peak at 24–48 h (*P* < 0·05). These changes in the population densities might be the result of quorum sensing mechanisms in the microbiota of the gut (Mukherjee and Bassler, 2019). In addition, this reflects the fact that initial colonization with aerobic bacteria contributes to the creation of an anaerobic environment, which eventually leads to the establishment of obligate anaerobes in the intestinal tract (Mackie *et al.* 1999; Favier *et al.* 2003). The results of the present study are also consistent with Ducluzeau's (1983) statement that the intestinal microbiota of the newborn mammal reaches maximum levels as early as 24 to 48 h after birth. In line with the observations of Smith (1965) on the development of the gastrointestinal microbiota of newborn calves, the counts of the investigated germ groups are also subject to a continuous change up to the 7th day after birth. Molecular biological studies suggest that the calf's bacterial composition changes continuously in the first 3 months after birth (Uyeno *et al.* 2010; Alipour *et al.* 2018). In view of the heterogeneity in the development of the bacterial counts between the calves, however, the following observations could be made: While there were still remarkable differences in the individual calves' bacterial counts at the first three sampling times (0, 6, 12 h), the quartile distance decreased clearly after 24 h. Evaluating the aerobic and anaerobic bacterial counts, no differences of the deviations of Q1 and Q3 in relation to the median (Q2) could be determined at the respective measuring points (data not shown). Another situation arises when the distribution of germ counts is considered more differentiated: In the case of *Enterobacteriaceae*, the differences between Q1 and Q3 in relation to Q2 are greatest at times 6 and 12 h postpartum (Q1/ Q2: 0·039 and 0·002, Q3/ Q2: 85·937 and 5·000, respectively; e.g. the corresponding 24 h values are 0·406 and 2·423, respectively). The values of enterococci were most pronounced between 0 and 12 h postpartum, whereas in lactobacilli it was up to 48 h postpartum (data not shown). This can be interpreted as the beginning of the stabilization of the gut microbiota.

In all meconium samples, values between 2 × 10^2^ and 4·2 × 10^7^ CFU per g faeces could be detected for aerobic bacteria, whereas the anaerobes were below the detection limit in six of the meconium samples. The maximum value of the anaerobes was 7·8 × 10^7^ CFU per g faeces in one meconium sample. No relevant data exist for meconium samples of calves. However, this observation coincides with the investigations of Ducluzeau (1983), who had determined bacterial counts between ‘not detectable’ and up to 10^8^ CFU per g in human meconium. While it has previously been assumed that the gastrointestinal tract of mammalian foetuses is sterile at birth (Mackie *et al.* 1999), there is recent evidence that a prenatal transfer of maternal bacteria may occur to the foetus (Jiménez *et al,* 2005; Jiménez *et al.* 2008; Alipour *et al.* 2018). However, since humans, mice and ruminants have a different placental structure, prenatal germ transmission might be different – if possible at all. In addition, none of the calves of the present study was born by caesarean section, so that a perinatal germ colonization of the neonate is likely, and a contamination of the samples cannot be completely excluded. Although the faecal samples were stored chilled immediately after collection, it is still possible for some bacteria to grow until the time of the examination (maximum 48 h).

Enterobacteria (with *E. coli* being their most common representative) and enterococci achieved high bacterial counts within the first 24 h after birth, but decreased again after reaching a peak at 48 h. The lactobacilli, on the other hand, developed more slowly, but continued to increase until the 7th day after birth and finally surpassed the enterococci. The observed bacterial counts and courses of bacterial counts of the present study broadly agree with those of other authors (Smith and Crabb 1961; Smith 1965; Vlková *et al.* 2008). This consistent development of the different bacterial genera could be related to the fact that the pH values of the gastric contents are still relatively high immediately after birth. This promotes the proliferation of bacteria, particularly those of the calf's environment like *E. coli* and *Enterococcus* spp., which colonize the small and large intestines in high amounts (Smith 1965). By lowering the gastric pH (approximately on the second day of life), the lactobacilli finally gain a selection advantage because they can grow at lower pH values. These bacteria subsequently become the dominant members of the stomach and small intestine microbiota, but also colonize the colon (Smith 1965). At the age of 20 weeks, the lactobacilli finally represent the dominant bacterial genus in all intestinal compartments, including caecum and colon (Vlkova *et al.* 2006).

A closer look at the enterobacteria revealed that the proportion of lactose‐positive germs was still below 40% at the time of birth, but increased to more than 80% of the total number of enterobacteria after 6 h already. A similar course results when comparing *E. coli* (which represents the major part of the lactose‐positive *Enterobacteriaceae* in the present study) with other enterobacteria. The lactose‐negative enterobacteria detected during the first sampling times were mostly germs of the genera *Pantoea*, followed by *Hafnia* and *Serratia*. As *Pantoea* spp., especially *P. agglomerans*, are associated with plants (Dutkiewicz *et al.* 2016), we presume primarily a contamination from the farm environment. Especially in newborns, allochthonous bacterial species can be detected, which normally occur in habitats other than the gastrointestinal tract (Mackie *et al.* 1999). Therefore, the identified lactose‐negative enterobacteria of the present study might only be intestinal passersby. The species identification of enterococci showed that both *E. faecalis* and representatives of the *E*. *nonfaecalis/nonfaecium* group could be detected regularly and in high numbers in the faeces of healthy calves. The occurrence of enterococci in the faeces of calves is well documented (Devriese *et al.* 1992). However, no data on their developmental kinetics – especially *E. faecalis* and *E. faecium *–‐ in the faeces of newborn calf are available in the literature. It is noteworthy that *E. faecium*, a species which is used as a probiotic for calves, could only be found infrequently and in low counts in our study, where only healthy calves were included.

MALDI‐TOF analysis of the colonies isolated from LAMVAB agar resulted in the detection of 36 different species. The predominant number of isolates (2039 out of 2388) could be identified as *Lactobacillus* spp). As confirmed by Gram staining, LAMVAB agar also allowed the growth of cocci which were identified as *Leuconostoc* spp., *Lactococcus* spp. and *Pediococcus* spp. by MALDI‐TOF‐MS analysis (Table 1). Particularly frequently, isolates of the early sampling times could not be assigned to any species, presumably because there were no corresponding entries in the database of the MALDI‐TOF‐MS device. Especially with regard to the question, which bacteria play a central role as primary settler, a further species analysis (preferably with molecular biological methods) would be extremely interesting. The development of the individual *Lactobacillus* spp. is subject to considerable variations. At the sampling times 0 and 6 h, *L. brevis and L. plantarum* were detected most frequently, but were outnumbered by other *Lactobacillus* species as early as 12 h postpartum. *L. paracasei* dominates at 12 and 24 h, but is replaced by *L. murinus* and *L. reuteri* after 48 h. Finally, on the 7th day after birth, the number of *L. reuteri* exceeds the number of all other detected lactobacilli species significantly (*P* < 0·05). The investigation of newborn piglets showed that the lactobacilli population is undergoing a continuous change and that the dominant *Lactobacillus* type is changing within the first week of life (Tannock 2001). Studies on lactobacilli populations of calves are rare. Soto *et al. *(2010) found *L. salivarius* as being the dominant species in six young calves (without a specification of age), while Busconi *et al. *(2008)) identified *L. mucosae* in two 65‐day‐old animals. In the present study, *L. salivarius and L. mucosae* were also detected within the first week of life (Table 2). However, our results demonstrate that *L. reuteri* is the dominant *Lactobacillus* sp. in the faeces of calves in the second half of the first week of life. This fact seems to be very important for the calf’s intestinal health as *L. reuteri* showed *in vitro* bactericidal effects against *Shigella* sp., enterovirulent *E. coli* and *Vibrio cholerae* (Liévin‐Le Moal and Servin 2014), as well as anti‐infective activities against rotavirus and *Cryptosporidium parvum* (Alak *et al.* 1997; Seo *et al.* 2010).

## Conflict of Interest

None declared.
